# Cut Throat

**Published:** 1909-09-11

**Authors:** 


### Laryngology and Rhinology.

### CUT THROAT.

Wounds of the larynx and trachea may be the result of gunshot injuries or of any form of accident, but they are most often self-inflicted and due to attempted suicide. There is, curiously enough, a widespread belief among would-be suicides that death follows rapidly on opening the windpipe, and consequently the head is usually thrown well back and the cut is made in or near the middle line of the neck. This position makes the thyroid cartilage and trachea very prominent and, as the muscles of the neck are generally strongly contracted at the same time, the great vessels are well protected by the sterno-mastoid muscles and the cartilages of the wind-pipe, and they often escape injury. Suicidal wounds are usually more or less horizontal and are naturally made from left to right, unless the subject be left-handed. The lesion is very frequently made above the hyoid bone, for there seems to be a general vague idea that cutting the throat in this position is a rapidly fatal procedure, but as a matter of fact such wounds merely enter the thickness of the lingual muscles and do not penetrate to the pharynx or do any very serious harm; the lingual artery of one or both sides may be divided, and hæmorrhage from it may be free.

Wounds made in the thyro-hyoid space frequently reach the pharynx and injure the epiglottis; this may be partially severed and may cause suffocation by hanging down over the aperture of the larynx. As the blood flows into the throat above the vocal cords, it can be coughed out and will not enter the lungs to cause asphyxia or pneumonia. The thyroid cartilage is hard and is rarely cut through, but it may be much hacked about, so that parts of it necrose later on. The larynx may be opened below the cords by a wound through the crico-thyroid membrane, but the upper part of the trachea, which is prominent in the neck, is a site very often selected for the cut. Here the windpipe is readily entered; sometimes the trachea is completely divided, when the lower end will retract from the wound deeply into the neck and produce a very dangerous dyspnœa. The œsophagus may also be opened and even the cervical vertebrae have been scored by the knife. The most severe wounds are usually inflicted by persons definitely insane and, as the great vessels are then also generally involved, they are very rapidly fatal.

The two most urgent sources of danger are hæmorrhage and asphyxia. If the great vessels have been opened, the patient is usually already dead before assistance arrives. Next to death from loss of blood, the most pressing danger is that of the entrance of blood into the trachea and lungs. Blood which enters below the vocal cords is expelled with difficulty, and this difficulty is increased by the fact that the patient is nearly always in a very depressed and collapsed condition. This blood either kills at once by suffocating the patient, or it later sets up the very fatal form of inflammation of the lungs known as "inhalation pneumonia." Shock is always a prominent feature, and the patient has generally no wish to live; still, if he survive the early dangers of shock and hæmorrhage, he may recover from very extensive injuries. Small penetrating wounds may produce the most extensive emphysema. The later dangers are those of sepsis and pneumonia. Asphyxia may be immediate, and due to retraction of the completely severed trachea, or it may come on later and be caused by inflammatory swelling, which ensues with great rapidity. Finally, cicatricial contraction may result, and cause most severe and intractable stenosis.

The first point, then, is to stop the bleeding; if blood is entering the wind-pipe, pressure must be at once applied, tracheotomy performed, and the trachea packed with gauze above the cannula. If the wound is low down in the trachea, the cannula may be bandaged round with gauze and inserted through the wound; but it is far better, whenever possible, to introduce the tube through a clean tracheotomy incision made below the wound. Any packing introduced into the air-passages should always be in the form of ribbon-gauze with no cut edges from which threads may be detached, and both ends of every piece of gauze must be fixed to a tape tied loosely round the neck, for the sucking action of respiration is very powerful, and a loose piece will be readily drawn down the trachea. If the hæmorrhage can be thoroughly checked, and no blood is entering the wind-pipe, it is open to question whether tracheotomy should, or should not, be performed. It may sometimes be avoided when the wound is clean and sharply incised, but it must be remembered that œdema may come on with great rapidity, that the bleeding may recur, and that, if the wound become septic, the discharge may find its way into the trachea and set up pneumonia. It is, therefore, advisable, in the large marjority of cases, to introduce a tracheotomy tube below the wound and to plug the wind-pipe above the cannula. It is most important, for the avoidance of subsequent stenosis, to secure very accurate co-aptation by suture of the wound in the air-passage. The muscles and fasciae of the neck should also be carefully sutured as far as may be permitted by due regard to the very free drainage required. It need hardly be said that, if the wound be very dirty or much contused, stitches should only be inserted where absolutely necessary; but it is in any case essential to sew up the cut in the wind-pipe, or otherwise intractable narrowing will certainly result; and this can be done without danger if a tracheotomy tube has been inserted lower down. Wounds of the œsophagus or pharynx must also be carefully sutured with a separate layer of stitches in the mucous membrane, and ample provision for drainage down to the wound. In such cases the patient must be fed by the rectum for about three days, unless a soft œsophageal tube can be retained *in situ*. In other cases there is no reason to withhold food by the mouth, but it is always wise to begin by giving a few spoonfuls of sterilised water, to make sure that it passes the wound safely. Finally, treatment must be directed to the accompanying shock; if blood has been aspirated, opium is dangerous, and stimulating expectorants are indicated.

